# A Computational Maxwell Solver for Nonlocal Feibelman Parameters in Plasmonics

**DOI:** 10.1021/acs.jpcc.4c07387

**Published:** 2025-01-23

**Authors:** Lorenz Huber, Ulrich Hohenester

**Affiliations:** Institute of Physics, University of Graz, Universitätsplatz 5, 8010 Graz, Austria

## Abstract

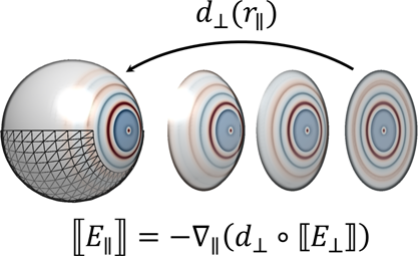

Feibelman parameters provide a convenient means to account for quantum surface effects in classical Maxwell descriptions. Recent work has shown that for incoming fields with spatial variations in the nanometer range, for instance those produced by quantum emitters in the vicinity of metallic nanoparticles, nonlocality in the directions parallel to the interface must be considered. Here we develop the methodology for mesoscopic boundary conditions incorporating nonlocal Feibelman parameters, and show how to implement them in a computational Maxwell solver based on the boundary element method. We compare our results with Mie solutions for single and coupled spheres, and find very good agreement throughout.

## Introduction

Science often makes progress in cycles. The first encounter with plasmonic nanoparticles goes back to the days of Zsigmondy and Mie around 1900,^1,2^ but it took until the 1960s until the basic physical mechanisms underlying plasmonics were further explored and understood.^3,4^ The advent and progress in the field of nanoscience and nanotechnology around 2000 triggered another cycle that finally brought plasmonics to its full glory.^5,6^ This plasmonics revolution is nicely captured in the perspective article “Plasmonics: an emerging field fostered by Nano Letters”^7^ by Naomi Halas, to be honored together with Peter Nordlander in this Festschrift. Not only she contributed over many years to the forefront of plasmonics research, but also promoted as the Editor-in-Chief of Nano Letters the full breadth of the field.

Peter Nordlander played a leading role in the field of plasmonics theory and simulations. Besides many other activities, he was always curious how quantum effects influence plasmonic behavior. In ref (8), he and co-workers investigated quantum tunneling in subnanometer gaps between plasmonic nanoparticles, using time-dependent density functional theory. However, he knew too well that the plasmonics community would start investigating such quantum effects only if they could be also simulated with classical Maxwell solvers. This led to the development of the so-called “quantum-corrected model”,^9,10^ which mimics electron tunneling by placing a fictitious medium with a finite conductivity in the gap region.

In the light of these achievements, we hope that the present study of nonlocal Feibelman parameters can raise the interest of the honored ones: it is based on a topic that has come to the spotlight in cycles, and it bridges between quantum descriptions and classical electrodynamic simulations. Feibelman parameters were first introduced in the 1970s in the field of surface science for the description of quantum effects in the vicinity of metal-dielectric interfaces.^11,12^ They were used later in plasmonics to estimate the spill out of conduction electrons^13^ and to introduce modified, so-called “mesoscopic boundary conditions”,^14,15^ which can be incorporated into classical Maxwell solvers. In the latter context, it has been demonstrated recently that for quantum emitters placed in the vicinity of plasmonic nanoparticles the Feibelman parameters should additionally account for nonlocality in the directions parallel to the metal surface.^16,17^

In this paper, we develop the methodology for mesoscopic boundary conditions incorporating nonlocal Feibelman parameters, and implement them into a computational Maxwell solver based on the boundary element method. Our approach follows earlier work^18^ where we implemented local Feibelman parameters into our NANOBEM toolbox.^19,20^

The manuscript has been organized as follows. In the Theory section we present the theory of nonlocal Feibelman parameters and sketch our numerical approach. In contrast to related work,^14,15,21^ we do not introduce the effect of Feibelman parameters through surface charge and current distributions, but rather follow the seminal works of Feibelman^11^ and Apell^22^ who directly started from Maxwell’s equations and then introduced appropriate surface response functions. As we will show below, only a slight twist is needed to render this approach suitable for the derivation of mesoscopic boundary conditions incorporating nonlocal Feibelman parameters, using a reasoning very similar to classical electrodynamics. Results for selected examples are presented next and are shown to be in very good agreement with Mie theory. Finally, we give a brief summary and an outlook to future work.

In this paper we solely investigate sodium nanospheres, which have received considerable interest in the literature because they can be also investigated using ab initio methods. The comparison with simplified description schemes, such as those based on Feibelman parameters, then allows us to evaluate the accuracy of the simplified schemes. Throughout we use the ab initio results presented in refs (16, 17) for benchmarking our approach. However, the implication of the very good agreement obtained in this work between the two approches is more far-reaching, as it suggests that quantum surface effects can be handled with equal success for the scientifically and technologically more interesting cases of noble metals and complex geometries using computational Maxwell solvers with runtimes that are comparable to those of classical solvers.

## Theory and Numerical Approach

### Theory

In refs (14, 23) the authors presented an approach based on Feibelman parameters^11,12^ to account for quantum effects close to the interface between a metal and a dielectric. They started with the frequency-dependent Feibelman parameters *d*_⊥_(ω), *d*_∥_(ω), which provide a measure of the distance over which the electric fields deviate from those for a sharp interface, and translated these parameters to modified boundary conditions. In the following, we adopt a slightly different approach. Following the reasoning of Feibelman^11^ and Apell,^22^ we start from the true microscopic description and obtain the mesoscopic boundary conditions using an approach very similar to classical electrodynamics.

**Figure 1:**
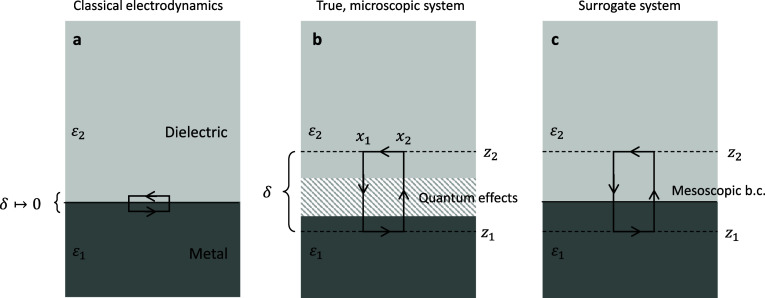
Schematics of Feibelman parameters. (a) In classical electrodynamics one assumes piecewise constant permittivities ε_1_, ε_2_ above and below the interface. For the tangential electromagnetic fields, the boundary conditions at the interface are obtained by integrating over a small surface area crossing the interface, and setting the height δ → 0. (b) Quantum effects close to an interface region *z* ∈ [*z*_1_, *z*_2_] are described in terms of the true, microscopic fields , . (c) In mesoscopic electrodynamics one seeks for a simplified description in terms of surrogate fields *E*, *H* and local permittivities ε_1_, ε_2_, where the modified boundary conditions are chosen such that outside the interface region ,  and *E*, *H* coincide. In the theoretical analysis the height of the surface area δ has to be set to a finite value, typically of the order of one nanometer.

We consider the setup depicted in Figure 1. Panel (b) shows the microscopic (“true”) system with electromagnetic fields , , whose dynamics in the vicinity of the interface depends on quantum effects in the metal, such as spill-out or reduced screening. The main assumption of our following analysis is that quantum effects are only important in a small region around the interface *z* ∈ [*z*_1_, *z*_2_], which is typically of the order of one nanometer (the actual choice of the region is arbitrary, it only must be sufficiently large), whereas outside the region the material response can be described in terms of the usual permittivities ε (we set all permeabilities to μ_0_ throughout). Our goal now is to introduce the surrogate system shown in panel (c) with electromagnetic fields *E*, *H*, whose dynamics away from the boundary can be described in terms of ε_1_, ε_2_ only, with no explicit consideration of quantum effects. We request that outside the interface region [*z*_1_, *z*_2_] the electromagnetic fields ,  and *E*, *H* coincide, which will be achieved by introducing modified boundary conditions for *E*, *H* to be presented below. With this, one can incorporate quantum surface effects into a classical electrodynamic description, and the electromagnetic fields of the classical approach correspond to those of the quantum approach outside the interface region.

We start to derive Maxwell’s boundary conditions similarly to classical electrodynamics^24^ by integrating Faraday’s law over a small rectangular surface area perpendicular to the interface and centered around *x* and *z* = 0 (see Figure 1),
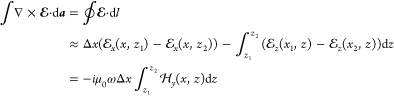
1Throughout we consider Maxwell’s equations in the frequency domain and a time dependence of the form e^–*i*ω*t*^. Because of quantum effects in the vicinity of the interface we have to keep *z*_1_, *z*_2_ finite, contrary to classical electrodynamics where δ can approach zero such that the integrals become zero, see Figure 1a. The integrals in eq 1 thus account for quantum corrections. As stated above, outside the interface region the true and surrogate fields coincide, therefore we set  and . For small differences *x*_2_ – *x*_1_ the term with the electric fields  under the integral can be expanded in a Taylor series around *x*, and we can thus rewrite eq 1 in the form
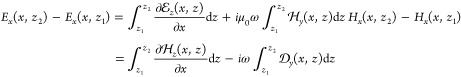
2Note that we have canceled the common factor Δ*x*. The expression in the second line of eq 2 can be obtained in analogy to the first one from Ampère’s law. We next introduce the deviation of the true field from the surrogate one, , with corresponding expressions for the other fields. The integral expressions in eq 2 are evaluated in terms of this decomposition, and we additionally perform a Taylor series expansion for the electromagnetic fields *E*, *H* around *z* = 0 to arrive at
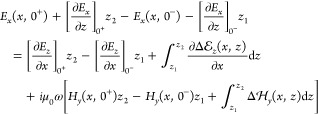
3where 0^+^ and 0^–^ indicate *z* values slightly above or below the interface. All terms in brackets either cancel each other or can be neglected. To see this, we express Faraday’s law for *E*, *H* in Cartesian coordinates
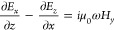
and observe that the terms in brackets of eq 3 which are proportional to *z*_1_ and *z*_2_ cancel out. As regarding the term with  under the integral, we first note that in classical electrodynamics *H*_*y*_ is a conserved quantity at the interface. The last term in eq 3 then corresponds to a small correction integrated over a a small region, which can be neglected if we are only interested in the leading order corrections to Maxwell’s boundary conditions. This point is discussed in more detail in refs (11, 22). With this, we are led to the modified boundary conditions incorporating quantum surface effects,
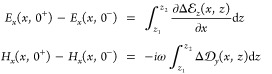
4The quantum corrections on the right-hand sides are proportional to the incoming surrogate fields and can be brought to a general form by introducing response functions, usually referred to as Feibelman parameters. There is some freedom of how to choose the response functions and we here follow refs (11, 22) who studied the reflection and transmission of an incoming plane wave with parallel wavevector ***k***_∥_ at a planar interface, and obtained (eqs 16, 18 of ref (22), see also the Appendix)
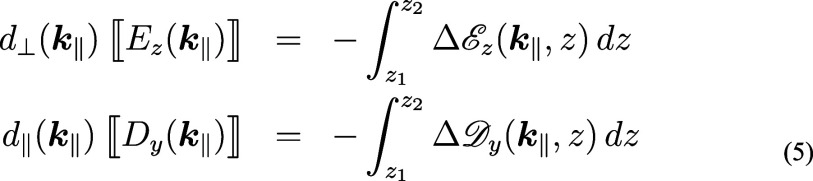
5Here we have introduced in accordance to ref (14) the shorthand notation 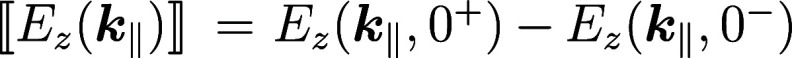 for the jump of *E*_*z*_ at the interface, with a corresponding expression for 
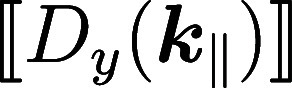
. When converting the above expressions from a wavevector representation ***k***_∥_ to a real-space representation ***r***_∥_ via a Fourier transform, the products in wavevector space on the left-hand side of eq 5 transform to convolutions in real space viz
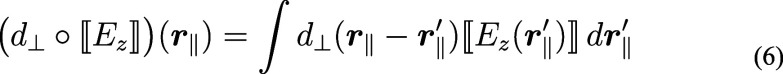
6with a corresponding expression for *D*_*y*_. With this, eq 4 can be brought to the final form of the mesoscopic boundary conditions (eqs 1c,d of ref (14))
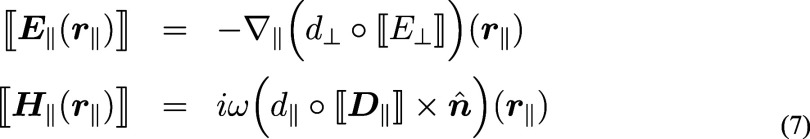
7***n̂*** is the outer surface normal of the interface, pointing from the metal to the dielectric, and we have used the notations *E*_⊥_, ***E***_∥_ for the normal and tangential components of the electric field, respectively. Note that in ref (14) the authors only considered local Feibelman parameters with *d*(***r***_∥_–***r***_∥_*′*) = *d*δ(***r***_∥_–***r***_∥_*′*).

### Numerical Implementation

In this section we provide details about the implementation of the nonlocal mesosocopic boundary conditions in our NANOBEM toolbox. We first discuss the working equations of the BEM approach, and then show how to convert the wavevector-dependent Feibelman parameters obtained from ab initio calculations to real-space representations.

The methodology of our BEM approach has been presented in some detail in refs (25, 26), see also ref (18) for a thorough discussion of the implementation using local Feibelman parameters. In the BEM approach, the particle boundary becomes discretized in terms of triangular boundary elements, and the tangential electric fields are expanded in terms of Raviart-Thomas shape elements ***f***_ν_(***r***_∥_) via (eq 11.36 of ref (26))
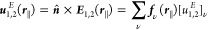
8with a corresponding expression for the magnetic field. The subscripts 1,2 label the fields inside and outside of the particle,  are the coefficients characterizing the tangential electric field, and ν is an index for the global degrees of freedom.

The representation formula (eq 5.27 of ref (26)) connects the electromagnetic fields at positions away from the boundary to the tangential electromagnetic fields on the boundary, eq 8. Upon letting the position approach the boundary, we obtain the so-called Calderon identities (eq 21 of ref (18))
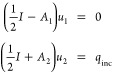
9Here *I* is the identity matrix, *A*_1,2_ are block matrices composed of single and double layer potentials (eq (18) of ref (18)),  is a vector formed by the field components, with a similar expression for *u*_2_, and *q*_inc_ is a vector for the incoming fields (eq (A3) of ref (18)). As described in more detail in ref (18), the boundary conditions of eq 7 can be brought to matrix form
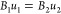
10where the matrices *B*_1_, *B*_2_ are given by eq A8 of the above-cited paper. When considering nonlocal Feibelman parameters, the submatrix *K* must be modified to
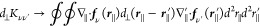
11with a corresponding modification for the matrix involving *d*_∥_. See also the Supporting Information for a more detailed analysis. The rest of our previous numerical approach can be kept without further modifications. Most importantly, the working equation of our BEM approach can again be obtained from the combination of the Calderon identities of eq 9 with the boundary conditions of eq 10,
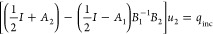
12For given incoming fields, this equation can be solved to obtain the unknown tangential fields *u*_2_ at the boundary.

### Feibelman Parameters

**Figure 2:**
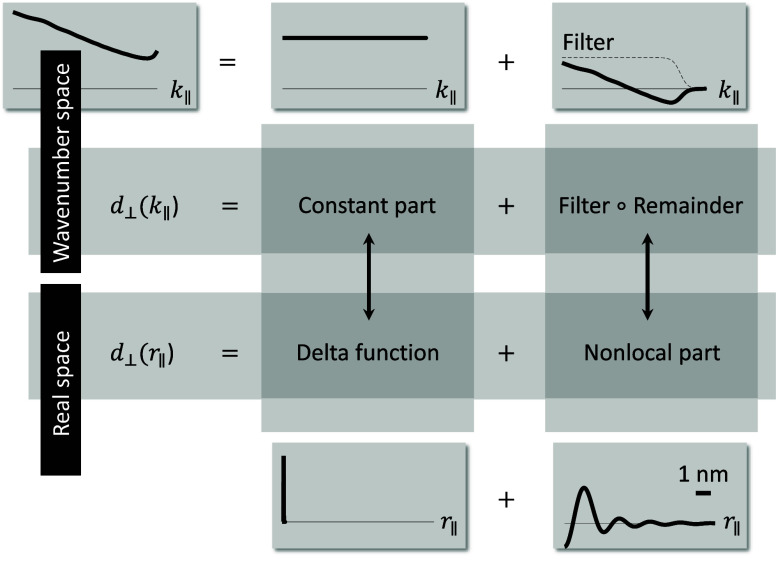
Conversion of nonlocal Feibelman parameters from wavenumber to real space. Ab-initio simulations for planar interfaces provide us with the wavenumber representation of the Feibelman parameters *d*_⊥_(*k*_∥_), which are decomposed into a constant part and a remainder. We use a filter function for the cutoff of the Feibelman parameters at large *k*_∥_ values. Upon Fourier transformation we obtain the real-space representation *d*_⊥_(*r*_∥_). The constant contribution translates to a delta function, and the filtered remainder to the nonlocal Feibelman parameters. The panels show results for sodium and a photon energy of 3.1 eV.^17^

Three steps are required to bring nonlocal Feibelman parameters into our BEM approach, see also Figure 2. The first and most important one concerns the computation of these parameters within an ab initio approach, such as time-dependent density functional theory (TDDFT). Typically one considers a slab geometry and obtains the parameters as a function of parallel wavenumber *k*_∥_ and light frequency. Details of the computation have been discussed elsewhere,^17^ and we here simply assume that the pertinent parameters are at hand.

In a second step, we bring the parameters into a form suitable for the transformation from wavenumber space to real space. Most importantly, we introduce a wavenumber cutoff *k*_cut_, which translates in real space to the neglect of short-range features. For incoming fields with no spatial dependence along the directions parallel to the interface, *d*(*k*_∥_ = 0) fully accounts for the average effect of Feibelman parameters, therefore a wavenumber cutoff is justified whenever the field modulations remain small on a length scale of .

In our approach we additionally split the Feibelman parameters into a constant contribution and a remainder, and perform for the latter part a cutoff using a smooth filter function, in order to avoid strong oscillations in the Fourier transformed function. The choice of the value for the constant term is somewhat arbitrary. From a physics point of view, the natural choice for the constant part would be *d*(*k*_∥_ = 0), as it corresponds to the local Feibelman paremeters. From a computational perspective, we observed that the average value of the parameters in the wavenumber space under consideration is usually better because it leads to functions that are smoother in real space, which can be integrated more easily.

The Fourier transformation from wavenmumber to real space is performed in a third step. As for the nonlocal part, we obtain
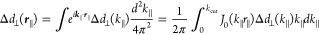
13where we have switched in the second line to polar coordinates and have integrated analytically over the azimuthal angle (assuming that the Feibelman parameters only depend on the wavevector modulus). Correspondingly, the constant part translates in real space to Dirac’s delta function, see Figure 2. In our computational approach, the constant part leads to local Feibelman parameters, which can be handled along the lines discussed in ref (18), whereas the remaining part leads to nonlocal Feibelman parameters, whose implementation has been discussed above.

## Results

In this section we present selected simulation results for single and coupled nanospheres, and for the decay rate modifications and energy shifts of quantum emitters placed in the vicinity of spheres. Our main goal is to demonstrate that quantum surface effects, which play an important role at small length scales, can be modeled accurately and efficiently by means of Feibelman parameters. As a proof-of-principle setup, we consider sodium spheres with a diameter of 7 nm only, for which ab initio results have been presented in the literature,^16,17^ but our approach can be easily to different material systems and more complex nanoparticle geometries (see for instance the Supporting Information where we show results for a sodium nanocube).

**Figure 3:**
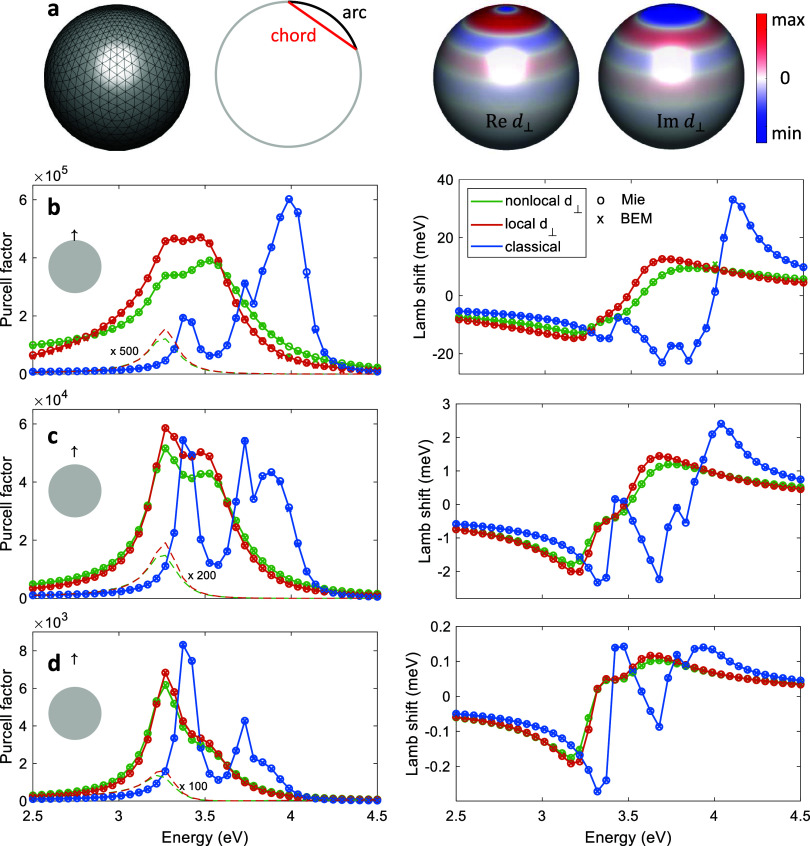
Dipole above sodium nanosphere. (a) We discretize the sphere boundary using a nonuniform mesh with a finer discretization in the vicinity of the position of the quantum emitter, here around the north pole. For the nonlocal Feibelman parameters, we use a parametrization in terms of the chord length, eq 19, as described in more detail in the text. The panels on the right show the real and imaginary part of *d*_⊥_(***r***_∥_–***r***_∥_*′*) (in arbitrary units) for a sphere with a diameter of 7 nm and for a photon energy of 3.3 eV. The source position ***r***_∥_ is located at the north pole. The other panels report the Purcell enhancement (left) and Lamb shift (right) for a sphere with a diameter with 7 nm, and for distances between the sphere and quantum emitter of (b) 1 nm, (c) 2 nm, and (d) 4 nm. The dipole moment is set in accordance to ref (17) to 0.1 e × nm, where *e* is the elementary charge, and the dipole points in the *z*-direction. We compare results obtained within classical Maxwell’s theory, and for mesoscopic boundary conditions incorporating local and nonlocal Feibelman parameters. The circle symbols report results of Mie calculations, the cross symbols results of our BEM simulations, and the dashed lines show the radiative enhancements that have been multiplied for better visibility by the factors reported in the insets. Throughout, we obtain very good agreement.

Throughout we use the nonlocal Feibelman parameters presented in ref (17), which were obtained from time-dependent density functional theory (TDDFT) simulations. We use for *d*_⊥_(***r***_∥_–***r***_∥_*′*) a parametrization in terms of the chord length, see Figure 3 and eq 19, in order to map the nonlocal Feibelman parameters obtained from ab initio simulations for flat interfaces to the sphere boundary. Instead of using for the parametrization the chord length, corresponding to the Euclidian distance, we could also take the arc length, which measures the distance along the nanoparticle boundary. Both approaches give similar results, and the investigation of the small differences and the optimal choice are left to future work. The Feibelman parameter *d*_∥_ is neglected but has been implemented into our simulation software and will be investigated in future work. In all our simulations we use sphere diameters of 7 nm and material parameters representative for sodium, with a Drude-type permittivity function.^17^ The dielectric constant of the embedding medium is set to one. For the local Feibelman parameters we use in agreement to ref (17) the results of ref (21).

Figure 3b–d show the Purcell enhancement (left) and Lamb shift (right) of a dipolar quantum emitter placed at distances of (b) 1 nm, (c) 2 nm, and (d) 4 nm above the sphere. In each plot we vary the transition energy of the dipole emitter, the dipole is assumed to point in the *z*-direction, and for the Lamb shift we use a dipole moment of 0.1*e* × nm, where *e* is the elementary charge. All quantities are defined in the same way as in ref (17).

We have developed a Mie theory including nonlocal Feibelman parameters, as discussed in more detail in the Appendix, and compare in Figure 3 the results of Mie theory and BEM simulations. Most importantly, we obtain very good agreement throughout. Let us briefly comment on the most important findings of the simulations. First, the Purcell factor obtained from classical electrodynamics without Feibelman parameters reflects the spectrum of plasmonic modes. In panel (d) one observes the dipolar (3.3 eV) and quadrupolar (3.7 eV) modes, together with a weak shoulder at higher energies associated with the pseudomode formed by all higher multipoles.^27,28^ Similarly, the Lamb shift reflects the usual frequency-dependent response of an oscillator when crossing one of its resonances. When the dipole is moved closer to the sphere, Figure 3b,c, it couples more efficiently to the evanescent fields of the plasmonic nanosphere, and the modes at higher energies are excited more strongly.

Things change significantly when considering Feibelman parameters. Here the modes broaden and red-shift, and throughout we only observe two distinct modes, associated with dipolar and quadrupolar resonances. Again the mode at higher energy becomes excited more strongly when the dipole approaches the nanosphere. When comparing the results for local and nonlocal Feibelman parameters, we observe that it is visible only for small dipole-sphere separations and nonlocality leads to an additional broadening of the Purcell enhancement and Lamb shift curves.

**Figure 4:**
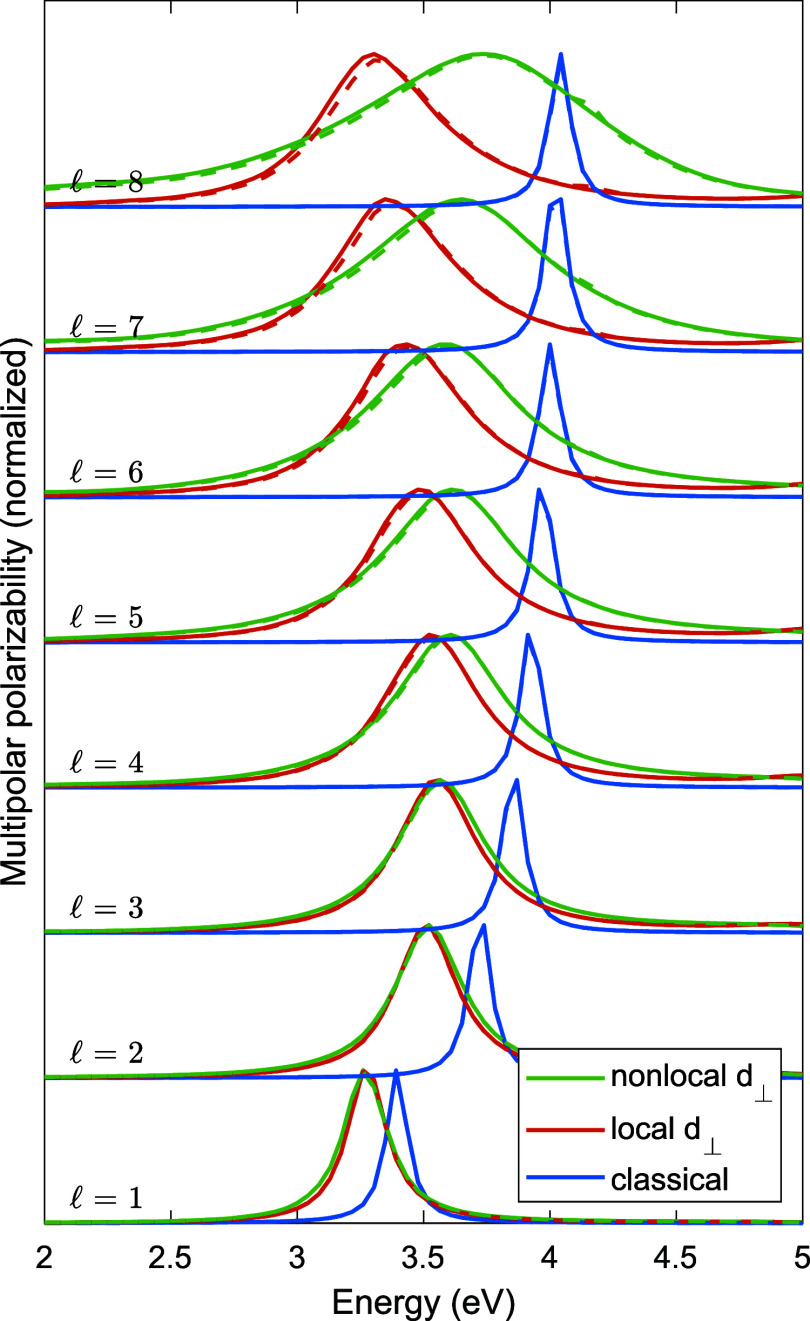
Multipole moments for sodium nanosphere (diameter 7 nm). We plot the imaginary part of the multipolar polarizability  as a function of transition energy, for details see text, and compare results for classical and mesoscopic boundary conditions, using either local or nonlocal Feibelman parameters. The solid and dashed lines report the results of Mie calculations and our BEM simulations, respectively.

The differences between local and nonlocal Feibelman parameters is investigated in more detail in Figure 4. There we plot the imaginary part of the multipolar polarizability , which we define in the same manner as in eq 32 of the SI of ref (21), and which accounts for the sphere response to a multipolar excitation with a well-defined angular degree . It is directly proportional to the transverse magnetic Mie coefficient . In our BEM approach,  can be obtained by using an excitation with a well-defined multipolar character and expanding the sphere’s response using a multipolar decomposition, see ref (29) for more details. In the figure, one observes that in the sphere’s response nonlocality plays a role for excitations where multipole orders above say three have to be considered.

**Figure 5:**
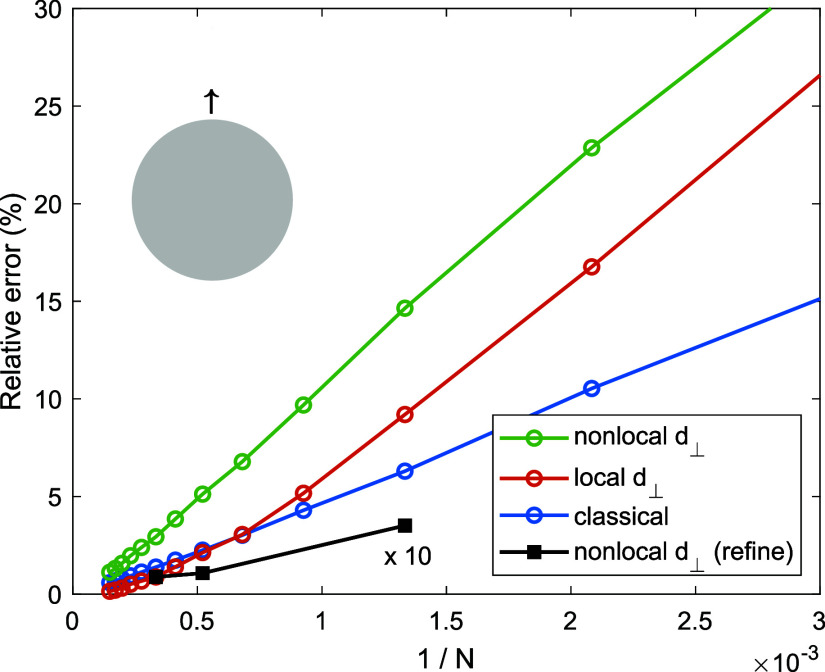
Convergence of BEM simulations. We show the relative error between results of Mie calculations and BEM simulations for the Purcell enhancement of a quantum emitter placed at a distance of 1 nm away from the sphere. The dipole transition energy is set to 3.5 eV. We use different sphere discretizations, with or without refinement at the north pole. *N* corresponds to the number of degrees of freedom in our BEM approach, this is the number of unique triangle edges.^18^ Note that the errors for the refined sphere discretization have been multiplied by a factor of 10 for better visibility.

In Figure 5 we investigate the convergence behavior of a dipole placed 1 nm above a nanosphere. We plot the relative error of the Purcell enhancement |γ_BEM_ – γ_Mie_|/γ_Mie_ for a transition energy of 3.5 eV and for different sphere discretizations. *N* is the number of unique triangle edges, which corresponds to the global degrees of freedom of our BEM simulations. The circles report the errors of simulation results for a uniform sphere discretization. We observe that the error monotonically decreases with increasing *N*, as expected for BEM implementations based on a Galerkin scheme.^30^ The black square symbols show results for a nonuniform discretization, where the mesh is refined at the north pole close to the dipole. Here the error is strongly reduced in comparison to simulations with a uniform mesh, with values below 1% even for relatively coarse discretizations.

**Figure 6:**
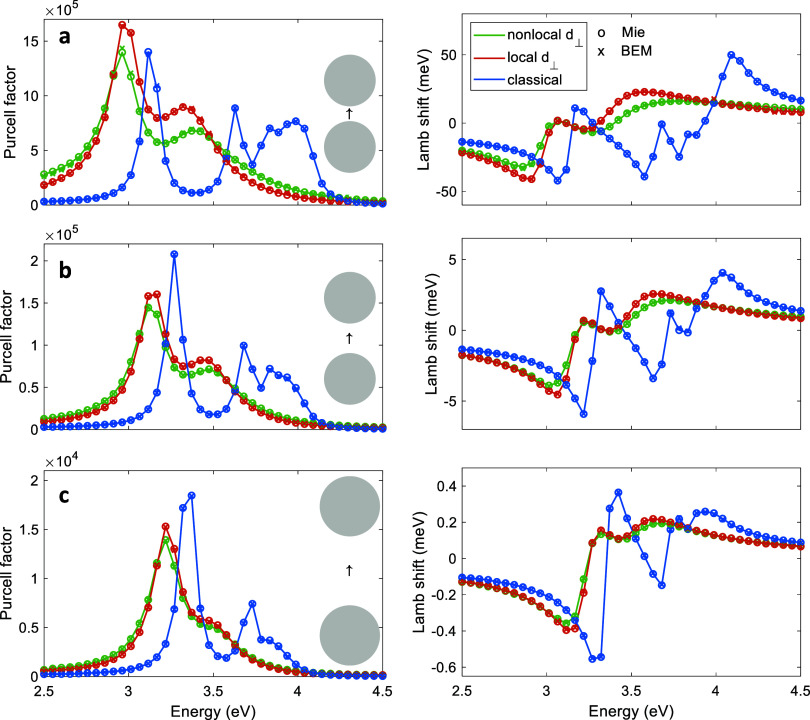
Same as Figure 3 but for dipole in gap region between sodium nanospheres. The spheres have diameters of 7 nm and the gap distances are (a) 2 nm, (b) 4 nm, and (c) 8 nm. In all simulations the dipole is oriented along *z* and located in the middle of the gap, as schematically shown in the insets.

Finally, in Figure 6 we show the Purcell enhancement (left) and the Lamb shift (right) for coupled nanospheres, using the same simulation parameters as for the single sphere shown in Figure 3. For the Mie theory, we compute the coupling coefficients for the spheres using the addition theorem.^31^ Again we observe almost perfect agreement between Mie theory and the BEM simulations. The Purcell enhancement factors can be interpreted in terms of the dimer modes, which now additionally contain bonding and antibonding modes, with a similar interpretation for the Lamb shift.

## Discussion and Summary

To summarize, in this paper we have developed the methodology for mesoscopic boundary conditions including Feibelman parameters that account for nonlocality in the directions parallel to a metal-dielectric interface, and have implemented them into a computational Maxwell solver based on the boundary element method. We have also presented a modified Mie theory including nonlocal Feibelman parameters. The results of Mie calculations and BEM simulations for dipolar quantum emitters interacting with single and coupled nanospheres have been shown to be in very good agreement throughout. We have demonstrated convergence of our simulation results, and have shown that nonuniform meshing around points where the electromagnetic fields exhibit large spatial variations can significantly increase the accuracy of the simulation results.

Currently our simulation code implemented in the NANOBEM toolbox is neither optimized for speed nor for efficiency. Runtimes depend on the simulation details, such as the size of the nanoparticle, the spatial cutoff for the nonlocal Feibelman parameters, or the length scale on which the parameters must be resolved. However, in general we observed that the runtimes for simulations with Feibelman parameters are about a factor of two to three slower than classical electrodynamics simulations, which constitutes no significant computational bottleneck. For this reason, we think that the appealing feature of Feibelman parameters is that they constitute a framework that is fully compatible with classical Maxwell solvers subject to minor modifications, in contrast to other schemes incorporating quantum effects, such as the hydrodynamic model, where substantial software developments are needed to combine them with electrodynamics simulations.

Quite generally, the NANOBEM toolbox works best for small to medium-sized problems with up to a few thousand degrees of freedom, where typical runtimes range from a few to several tens of minutes on a normal desktop computer. We thus think that BEM provides an ideal workbench for the investigation of mesoscopic boundary conditions and nonlocality for a wide range of plasmonic nanoparticles. There exist a number of concepts to perform BEM simulations also for significantly larger systems, using for instance hierarchical matrices,^32,33^ but we do not plan to go in this direction in the future. Rather we suggest to compute the T-matrices for single plasmonic nanoparticles, which can be done in complete analogy to ref (29), and to compute the optical response of coupled particles, particle clusters, or periodic particle arrays using one of the many T-matrix programs available, such as SMUTHI^34^ or TREAMS.^35^

An important future step will also be the detailed analysis of different geometries, including particles situated on substrates or layer structures, and the consideration of Feibelman parameters for different material combinations. In collaboration with other groups we plan to obtain the nonlocal Feibelman parameters from ab initio simulations, and to set up a database. This would make Feibelman parameters accessible to a broader community, and would establish mesoscopic boundary conditions as an integral part of plasmonics simulations.

## Appendix

### Comparison with Apell

In this Appendix we show that the definition of the Feibelman parameters in eq 5 is in accordance with the work of Apell.^22^ In this work he assumes that a plane wave with the parallel wavevector component *k*_*x*_ impinges on the interface at *z* = 0, and introduces for the electric field on the metal side the expressions (eq 3 of ref (22))

14

The normal wavevector component on the metal side is denoted with *k*_*z*_ (*p*_*t*_ in ref (22)), and the superscript *t* indicates that the fields are purely transverse. Let us consider the Feibelman parameter *d*_∥_ first, which is found to be of the form (eqs 6, 16 of ref (22))

15

Note that we have slightly adapted the prefactor because of the SI units adopted in this work, and use  rather than  because of the geometry for the incoming wave introduced in eq 14. Rearranging the terms brings us to

16

where we have assumed that on the left-hand side *E*_*x*_ is continuous when crossing the interface, which is justified when considering the effect of Feibelman parameters in lowest order only. The analysis for the perpendicular component of the Feibelman parameters is very similar. Our starting expression is (eqs 10, 18a of ref (22))

17

For the usual boundary conditions of Maxwell’s equations the normal component of the dielectric displacement is continuous when crossing the interface, which gives ε_0_*E*_*z*_(0^–^) = ε *E*_*z*_(0^+^). We thus get in the lowest order of Feibelman parameters

18

We finally note that, in contrast to the setup in Figure 1, Apell considers the situation where the orientation of the dielectric and the metal is exchanged. However, as can be seen by simultaneously changing the sign of  and  the definition of the Feibelman parameters is independent of the orientation.

### Mie Theory

In this Appendix we briefly sketch the steps needed for the implementation of nonlocal Feibelman parameters within Mie theory. For simplicity we only consider *d*_⊥_. For a spherical particle with radius *R*, the chord length between two points on the sphere is

19

where γ is the angle between ***r***_∥_ and ***r***_∥_*′*. As the distance is uniquely determined by the angle γ, we can expand the nonlocal Feibelman parameter using the complete set of Legendre polynomials viz

20

where we have used the addition theorem to arrive at the last expression (eq 3.62 of ref (24)). Eq 20 can be also used to compute the Feibelman coefficients . One further observes that for coefficients that don’t depend on the angular degree  one obtains the local contribution *R*^–2^*d*^⊥^δ(***r̂***_∥_–***r̂***_∥_*′*).

With the nonlocal Feibelman parameters defined in eq 20 we can set up Mie theory in close analogy to ref (21), with the main difference that the Feibelman parameters  additionally depend on the angular order. For the transverse magnetic Mie coefficient we then obtain in accordance to (eq 30a of SI of ref (21))

21

Herer ε_1_, ε_2_ are the permittivities of the sphere and the embedding medium, respectively, with corresponding parameters *x*_1_ = *k*_1_*R*, *x*_2_ = *k*_2_*R*,  and  are the spherical Bessel and Hankel functions, and ,  the usual Riccati-Bessel functions (eq E.11 of ref (26)). Upon neglect of  the transverse electric Mie coefficient is identical to the coefficient from classical Mie theory.

## Supporting Information Available

The Supporting Information contains more details about the implementation of the nonlocal Feibelman parameters in our boundary element method approach, as well as simulation results for a sodium nanocube. This research was funded in whole, or in part, by the Austrian Science Fund (FWF) 10.55776/P37150 (PDF)

## References

[ref1] Zsigmondy R. Über wässrige Lösungen metallischen Goldes. Justus Liebigs Ann. Chem. 1898, 301, 29 10.1002/jlac.18983010104.

[ref2] Mie G. Beiträge zur Optik trüber Medien, speziell kolloidaler Metallösungen. Annalen der Physik 1908, 330, 377 10.1002/andp.19083300302.

[ref3] Raether H. Surface Plasmons on Smooth and Rough Surfaces and on Gratings; Springer Tracts in Modern Physics; Springer: Berlin, 1988; Vol. 111.

[ref4] Kreibig U.; Vollmer M. Optical properties of metal clusters; Springer series in material science; Springer: Berlin, 1995; Vol. 25.

[ref5] Barnes W. L.; Dereux A.; Ebbesen T. W. Surface plasmon subwavelength optics. Nature 2003, 424, 824 10.1038/nature01937. 12917696

[ref6] Maier S. A. Plasmonics: Fundamentals and Applications; Springer: Berlin, 2007.

[ref7] Halas N. Plasmonics: an emerging field fostered by Nano Letters. Nano Lett. 2010, 10, 3816 10.1021/nl1032342. 20853888

[ref8] Zuloaga J.; Prodan E.; Nordlander P. Quantum description of the plasmon resonances of a nanoparticle dimer. Nano Lett. 2009, 9, 887 10.1021/nl803811g. 19159319

[ref9] Esteban R.; Borisov A. G.; Nordlander P.; Aizpurua J. Bridging quantum and classical plasmonics with a quantum-corrected model. Nat. Commun. 2012, 3, 825 10.1038/ncomms1806. 22569369

[ref10] Zhu W.; Esteban R.; Borisov A. G.; Baumberg J. J.; Nordlander P.; Lezec H. J.; Aizpurua J.; Crozier K. B. Quantum mechanical effects in plasmonic structures with subnanometre gaps. Nat. Commun. 2016, 7, 11495 10.1038/ncomms11495. 27255556 PMC4895716

[ref11] Feibelman P. J. Surface electromagnetic fields. Prog. Surf. Sci. 1982, 12, 287 10.1016/0079-6816(82)90001-6.

[ref12] Liebsch A. Electronic excitations at metal surfaces; Physics of solids and liquids; Springer: New York, 1997.

[ref13] Teperik T. V.; Nordlander P.; Aizpurua J.; Borisov A. G. Robust Subnanometric Plasmon Ruler by Rescaling of the Nonlocal Optical Response. Phys. Rev. Lett. 2013, 110, 263901 10.1103/PhysRevLett.110.263901. 23848876

[ref14] Yang Y.; Zhu D.; Yan W.; Agarwal A.; Zheng M.; Joannopoulos J. D.; Lalanne P.; Christensen T.; Berggren K. K.; Soljacic M. A general theoretical and experimental framework for nanoscale electromagnetism. Nature 2019, 576, 248 10.1038/s41586-019-1803-1. 31827292

[ref15] Mortesen N. A. Mesoscopic electrodynamics at metal surfaces. Nanophotonics 2021, 10, 2563 10.1515/nanoph-2021-0156.

[ref16] Babaze A.; Ogando E.; Stamatopoulou P. E.; Tserkezis C.; Mortensen N. A.; Aizpurua J.; Borisov A. G.; Esteban R. Quantum surface effects in the electromagnetic coupling between a quantum emitter and a plasmonic nanoantenna: time-dependent density functional theory vs. semiclassical Feibelman approach. Opt. Express 2022, 30, 21159 10.1364/OE.456338. 36224842

[ref17] Babaze A.; Neuman T.; Esteban R.; Aizpurua J.; Borisov A. G. Dispersive surface-response formalism to address nonlocality in extreme plasmonic field confinement. Nanophotonics 2023, 12, 3277–3289. 10.1515/nanoph-2023-0178. 39634140 PMC11501702

[ref18] Hohenester U.; Unger G. Nanoscale electromagnetism with the boundary element method. Phys. Rev. B 2022, 105, 075428 10.1103/PhysRevB.105.075428.

[ref19] Hohenester U.; Reichelt N.; Unger G. Nanophotonic resonance modes with the nanobem toolbox. Comput. Phys. Commun. 2022, 276, 108337 10.1016/j.cpc.2022.108337.

[ref20] Hohenester U. Nanophotonic resonators in stratified media with the nanobem toolbox. Comput. Phys. Commun. 2024, 294, 108949 10.1016/j.cpc.2023.108949.

[ref21] Goncalves P. A. D.; Christensen T.; Rivera N.; Jauho A.-P.; Mortensen N. A.; Soljcic M. Plasmon-emitter interactions at the nanoscale. Nat. Commun. 2020, 11, 366 10.1038/s41467-019-13820-z. 31953379 PMC6969188

[ref22] Apell P. A simple derivation of the surface contribution to the reflectivity of a metal, and its use in the van der Waals interaction. Phys. Scr. 1981, 24, 795 10.1088/0031-8949/24/4/019.

[ref23] Christensen T.; Yan W.; Jauho A.-P.; Soljacic M.; Mortensen N. A. Quantum Corrections in Nanoplasmonics: Shape, Scale, and Material. Phys. Rev. Lett. 2017, 118, 157402 10.1103/PhysRevLett.118.157402. 28452500

[ref24] Jackson J. D. Classical Electrodynamics; Wiley: New York, 1999.

[ref25] Chew W. C. Waves and fields in inhomogeneous media; IEEE Press: Picsatoway, 1995.

[ref26] Hohenester U. Nano and Quantum Optics; Springer: Cham, Switzerland, 2020.

[ref27] Delga A.; Feist J.; Bravo-Abad J.; Garcia-Vidal F. Theory of strong coupling between quantum emitters and localized surface plasmons. J. Opt. 2014, 16, 114018 10.1088/2040-8978/16/11/114018.

[ref28] Neuman T.; Esteban R.; Casanova D.; Garcia-Vidal F.; Aizpurua J. Coupling of molecular emitters and plasmonic cavities beyond the point-dipole approximation. Nano Lett. 2018, 18, 2358 10.1021/acs.nanolett.7b05297. 29522686

[ref29] Asadova N.; Achouri K.; Arjas K.; Auguie B.; Aydin R.; Baron A.; Beutel D.; Bodermann B.; Boussaoud K.; Burger S.; Choi M.; Czajkowski K. M.; Evlyukhin A. B.; Fazel-Najafabadi A.; Fernandez-Corbaton I.; Garg P.; Globosits D.; Hohenester U. T-matrix representation of optical scattering response: Suggestion for a data format. J. Quant. Spectrosc. Radiat. Transfer 2025, 333, 109310 10.1016/j.jqsrt.2024.109310.

[ref30] Buffa A.; Hiptmair R.; von Petersdorff T.; Schwab C. Boundary Element Methods for Maxwell Transmission Problems in Lipschitz Domains. Numer. Math. 2003, 95, 459 10.1007/s00211-002-0407-z.

[ref31] Bruning J. D.; Lo Y. T. Multipole scattering of EM waves by spheres Part I–Multipole expansion and ray-optical simulations. IEEE Trans. Antennas Propag. 1971, 19, 378 10.1109/TAP.1971.1139944.

[ref32] Hackbusch W. A sparse matrix arithmetic based on H-matrices Part I: Introduction to H-matrices. Computing 1999, 62, 89 10.1007/s006070050015.

[ref33] Börm S.; Grasedyck L.; Hackbusch W. Introduction to hierarchical matrices with applications. Engineering analysis with boundary elements 2003, 27, 405 10.1016/S0955-7997(02)00152-2.

[ref34] Egel A.; Krzysztof; Czajkowski M.; Theobald D.; Ladutenko K.; Kuznetsov A. S.; Pattelli L. SMUTHI: A python package for the simulation of light scattering by multiple particles near or between planar interfaces. J. Quant. Spectrosc. Radiat. Transfer 2021, 273, 107846 10.1016/j.jqsrt.2021.107846.

[ref35] Beutel D.; Fernandez-Corbaton I.; Rockstuhl C. treams–a T-matrix-based scattering code for nanophotonics. Comput. Phys. Commun. 2024, 297, 109076 10.1016/j.cpc.2023.109076.

