# Supplementary material for: A Computational Maxwell Solver for Nonlocal Feibelman Parameters in Plasmonics
Source: J Phys Chem C Nanomater Interfaces. 2025 Jan 23;129(5):2590–8. doi: 10.1021/acs.jpcc.4c07387 (PMC11808787; doi:10.1021/acs.jpcc.4c07387)
Supplement: Supplementary file 1 — jp4c07387_si_001.pdf [file jp4c07387_si_001.pdf]

A computational Maxwell solver for nonlocal Feibelman parameters in plasmonics

Lorenz Huber and Ulrich Hohenester*

Institute of Physics, University of Graz,

Universitätsplatz 5, 8010 Graz, Austria

In the Supplementary Information we provide more details about our BEM implementation and show additional simulation results for a sodium nanocube.

4 pages S1–S4

1 figures S1

0 tables

* E-mail `ulrich.hohenester@uni-graz.at`.

Nonlocal Feibelman parameters in BEM

In this section we provide additional information about our implementation of nonlocal Feibelman parameters in BEM. Our approach closely follows [1, 2], and we refer to this work whenever needed. As stated in the main text, the global degrees of freedom of the BEM approach are the tangential electromagnetic fields $\boldsymbol{u}_{1,2}$ inside and outside the nanoparticle. As regarding the Calderon identities of Eq. (9) of the main text, the derivation is identical to that of [2, Eq. (8)]. In the following we concentrate on the first boundary condition of Eq. (7) of the main text, which we repeat here for clarity

$$\llbracket \boldsymbol{E}_\parallel(\boldsymbol{r}_\parallel) \rrbracket = -\nabla_\parallel \Big(d_\perp \circ \llbracket E_\perp \rrbracket \Big)(\boldsymbol{r}_\parallel) \,. \tag{S1}$$

Ampère's law $\nabla \times \boldsymbol{H}_j = -i\varepsilon_j \omega \boldsymbol{E}_j$ enables us to express the normal component $E_j^\perp$ as

$$E_j^\perp = \hat{\boldsymbol{n}} \cdot \boldsymbol{E}_j = \frac{i}{\varepsilon_j \omega} \hat{\boldsymbol{n}} \cdot \nabla \times \boldsymbol{H}_j = -\frac{i}{\varepsilon_j \omega} \nabla_\parallel \cdot \boldsymbol{u}_j^H \,, \tag{S2}$$

where we have rearranged the triple product in the last step. Recall that $\hat{\boldsymbol{n}}$ is the outer surface normal pointing from medium 1 to 2. Premultiplying Eq. (S1) with $\hat{\boldsymbol{n}}\times$ leads us to

$$\llbracket \boldsymbol{u}^E(\boldsymbol{r}_\parallel) \rrbracket = \frac{i}{\omega}\, \hat{\boldsymbol{n}} \times \nabla_\parallel \left(d_\perp \circ \nabla_\parallel \cdot \left[\frac{\boldsymbol{u}_2^H}{\varepsilon_2} - \frac{\boldsymbol{u}_1^H}{\varepsilon_1} \right] \right) (\boldsymbol{r}_\parallel) \,. \tag{S3}$$

To render this expression suitable for use within BEM, we approximate the tangential electromagnetic fields using the Raviart-Thomas shape elements $\boldsymbol{f}_\nu(\boldsymbol{r}_\parallel)$ via [1, Eq. (11.36)]

$$\boldsymbol{u}_j^E(\boldsymbol{r}_\parallel) = \sum_\nu \boldsymbol{f}_\nu(\boldsymbol{r}_\parallel) \left[u_j^E \right]_\nu \,, \tag{S4}$$

with a corresponding expression for $\boldsymbol{u}_j^H$. Additionally, in the Galerkin scheme adopted here we multiply Eq. (S3) from the left-hand side with the same shape functions and integrate over the particle boundary to arrive at

$$\begin{aligned} &\sum_{\nu'} \left(\oint \boldsymbol{f}_\nu(\boldsymbol{r}_\parallel) \cdot \boldsymbol{f}_{\nu'}(\boldsymbol{r}_\parallel)\, d^2 r_\parallel \right) \left[u_{2\nu'}^E - u_{1\nu'}^E \right] \\ &\quad = \frac{i}{\omega} \left(\oint \oint \boldsymbol{f}_\nu(\boldsymbol{r}_\parallel) \cdot \hat{\boldsymbol{n}} \times \nabla_\parallel d_\perp(\boldsymbol{r}_\parallel - \boldsymbol{r}_\parallel') \nabla_\parallel \cdot \boldsymbol{f}_{\nu'}(\boldsymbol{r}_\parallel')\, d^2 r_\parallel d^2 r_\parallel' \right) \left[\frac{u_{2\nu'}^H}{\varepsilon_2} - \frac{u_{1\nu'}^H}{\varepsilon_1} \right] . \end{aligned} \tag{S5}$$

The expression in parentheses on the left-hand side has previously been denoted with $J_{\nu\nu'}$ [2, Eq. (A5)]. In the expression in parentheses on the right-hand side, we perform integration by parts [2, Eq. (13)] to arrive at

$$\mathcal{I}_{\nu\nu'} = \oint \oint \Big(\nabla_\parallel \cdot \hat{\boldsymbol{n}} \times \boldsymbol{f}_\nu(\boldsymbol{r}_\parallel) \Big) d_\perp(\boldsymbol{r}_\parallel - \boldsymbol{r}_\parallel') \Big(\nabla_\parallel \cdot \boldsymbol{f}_{\nu'}(\boldsymbol{r}_\parallel') \Big)\, d^2 r_\parallel d^2 r_\parallel' \,. \tag{S6}$$

This expression is already very close to Eq. (11) of the main text. However, in accordance to [2] we face the problem that $\nabla_\parallel \cdot \hat{\boldsymbol{n}} \times \boldsymbol{f}_\nu$ is not at hand in our BEM approach and must be related to $\nabla_\parallel \cdot \boldsymbol{f}_{\nu'}$. Within the Galerkin scheme, the shape functions $\boldsymbol{f}_\nu$ provide a basis for representing the tangential vector fields. For this reason, we can express $\hat{\boldsymbol{n}} \times \boldsymbol{f}_\nu$ also in terms of these basis functions via

$$\hat{\boldsymbol{n}} \times \boldsymbol{f}_\nu(\boldsymbol{r}_\parallel) = \sum_{\nu''} C_{\nu\nu''} \boldsymbol{f}_{\nu''}(\boldsymbol{r}_\parallel)\,. \tag{S7}$$

Taking the inner product of this expression with $\boldsymbol{f}_{\nu'}$ then leads us to

$$\underbrace{\oint \hat{\boldsymbol{n}} \times \boldsymbol{f}_\nu(\boldsymbol{r}_\parallel) \cdot \boldsymbol{f}_{\nu'}(\boldsymbol{r}_\parallel)\, d^2 r_\parallel}_{-I_{\nu\nu'}} = \sum_{\nu''} C_{\nu\nu''} \underbrace{\oint \boldsymbol{f}_{\nu''}(\boldsymbol{r}_\parallel) \cdot \boldsymbol{f}_{\nu'}(\boldsymbol{r}_\parallel)\, d^2 r_\parallel}_{J_{\nu''\nu'}}\,. \tag{S8}$$

With this, we can bring Eq. (S6) to its final form

$$\mathcal{I}_{\nu\nu'} = -\left(I J^{-1} K\right)_{\nu\nu'}\,, \tag{S9}$$

where we have introduced in accordance to Eq. (11) of the main text the matrix K with the elements

$$K_{\nu\nu'} = \oint \oint \Big(\nabla_\parallel \cdot \boldsymbol{f}_\nu(\boldsymbol{r}_\parallel)\Big) d_\perp(\boldsymbol{r}_\parallel - \boldsymbol{r}'_\parallel) \Big(\nabla'_\parallel \cdot \boldsymbol{f}_{\nu'}(\boldsymbol{r}'_\parallel)\Big)\, d^2 r_\parallel d^2 r'_\parallel\,, \tag{S10}$$

Purcell enhancememt for sodium nanosphere

In the main text we have only considered spheres and coupled spheres, for which analytic and semi-analytic expressions can be obtained within Mie theory, in order to demonstrate the accuracy of our BEM approach. However, with the machinery developed we can also easily compute the response of other nanoparticle geometries.

As one example, in Fig. S1 we consider a sodium nanocube with a side length of 5 nm and dipoles located 1 nm above the cube, at the locations indicated in the right panel, with dipole orientations along z. In our BEM approach, we use a discretization with about 5800 triangles, and checked that the results are well converged for such as fine mesh. The Purcell enhancement for the three dipole locations is reported in the main panel. One observes that at the different dipole positions different plasmon modes can be excited, which can be associated with selected edge and face modes. More details will be presented elsewhere. For our present purpose we mainly would like to emphasize that the computational machinery

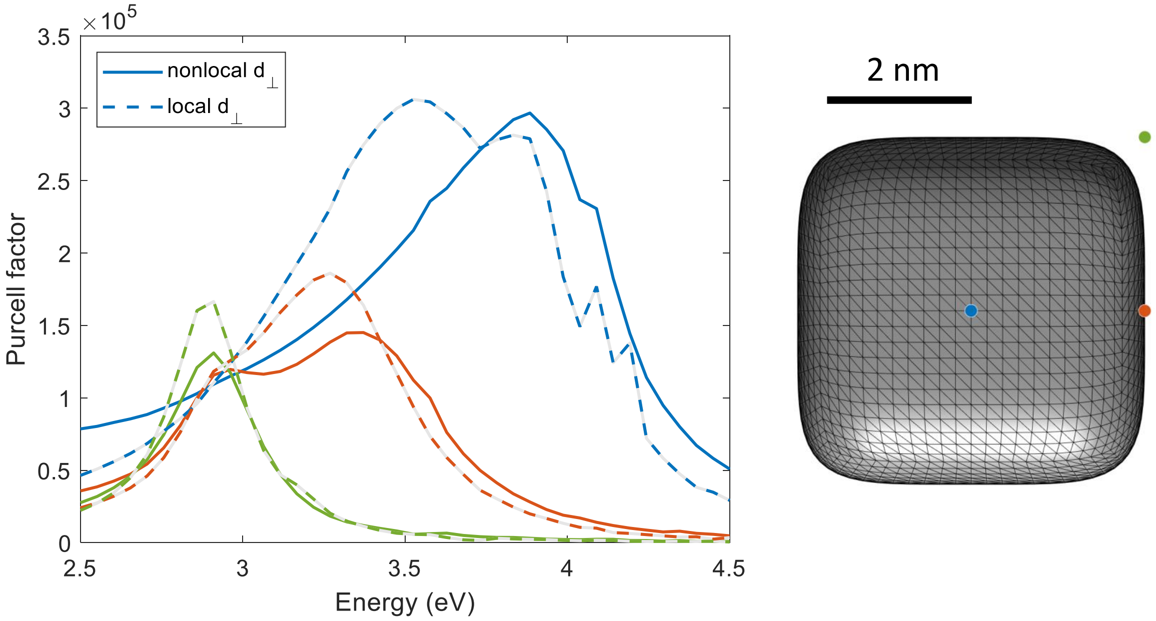

FIG. S1. Purcell enhancement for sodium nanocube. We consider a nanocube with a side length of 5 nm and dipoles located at the positions indicated in the right panel placed 1 nm above the the cube, with dipole orientations along z.

developed here is sufficiently versatile to be used for different geometries, including coated and coupled particles, as well as particles with arbitrary boundaries.

[1] U. Hohenester, *Nano and Quantum Optics* (Springer, Cham, Switzerland, 2020).

[2] U. Hohenester and G. Unger, Phys. Rev. B **105**, 075428 (2022).
